# Supplementary material for: Physiological, Biochemical, and Transcriptomic Responses of Neolamarckia cadamba to Aluminum Stress
Source: Int J Mol Sci. 2020 Dec 17;21(24):9624. doi: 10.3390/ijms21249624 (PMC7767006; doi:10.3390/ijms21249624)
Supplement: Supplementary file 1 [file ijms-21-09624-s001.pdf]

International Journal of
Molecular Sciences

MDPI

Supplementary data

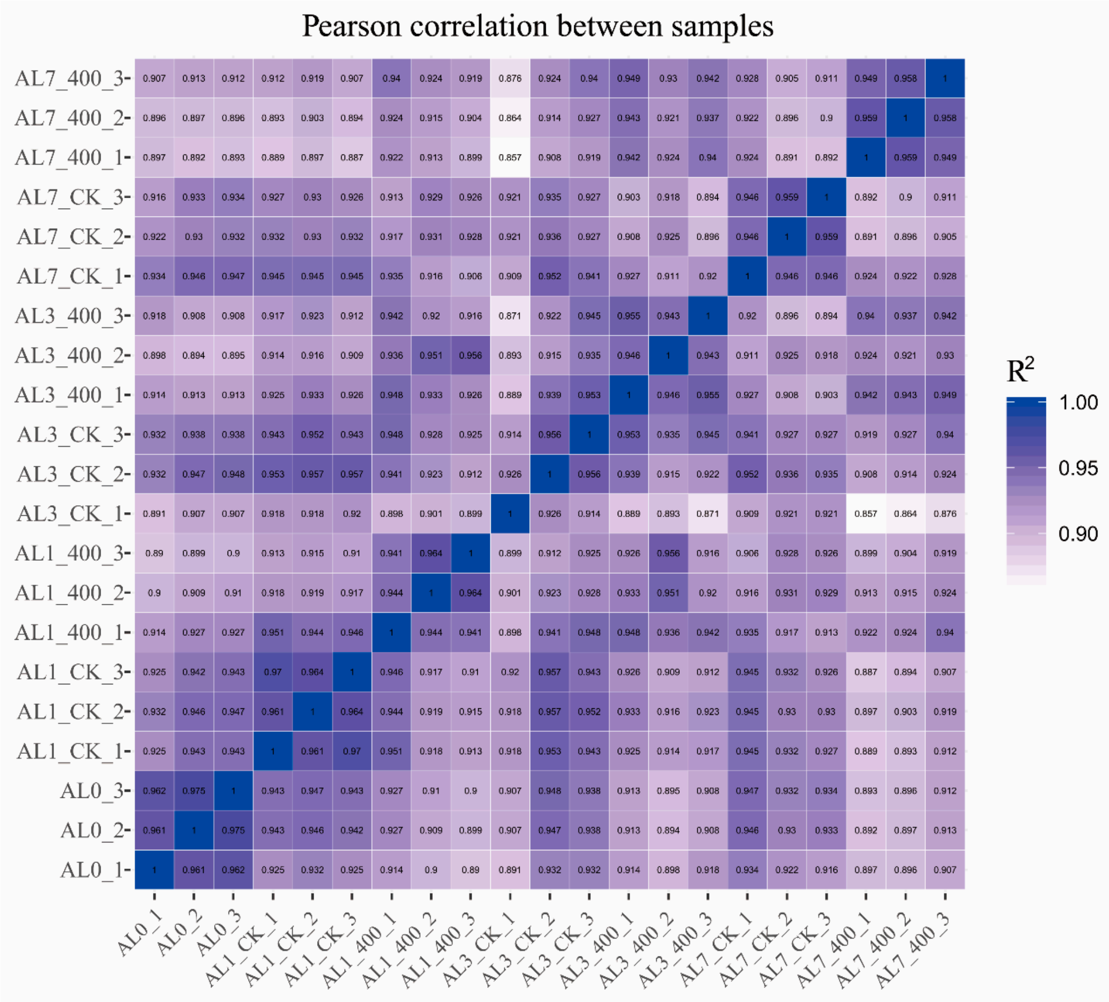

Figure Supplementary 1. Pearson correlation between samples. Taking the square of the Pearson correlation coefficient (R2) greater than 0.92 (under ideal sampling and experimental conditions), in specific project operations, R2 is required to be at least greater than 0.8 as the test index for the correlation of gene expression levels between samples, thereby verifying the reliability of the experiment and strictness of sample selection. The closer the correlation coefficient is to 1, the higher the similarity of the expression patterns between samples. The abscissa and ordinate are the log10 (FPKM+1) of the compared samples, and the ordinate is the sample type.

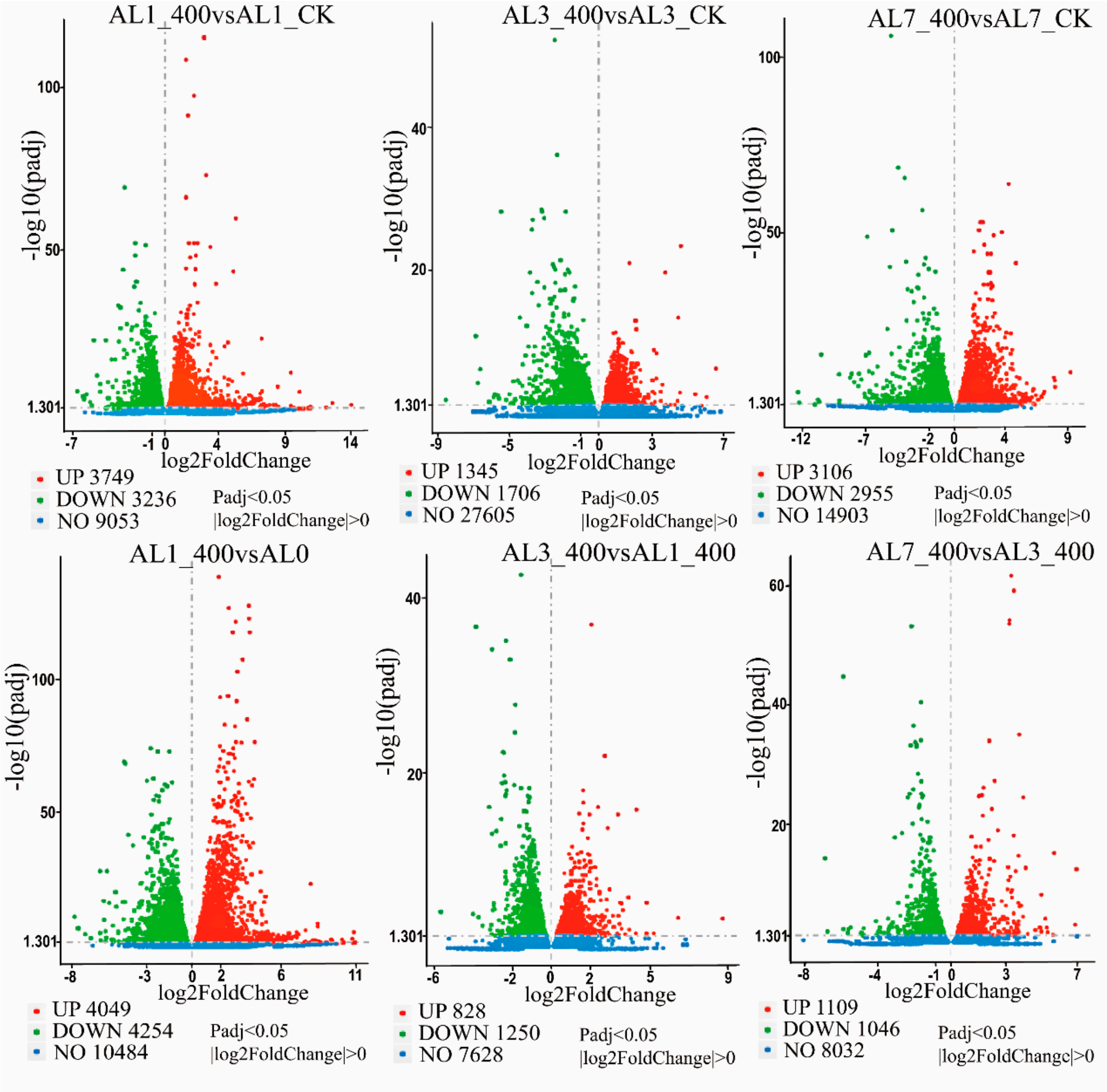

Figure Supplementary 2. Volcanic map of differentially expressed gene (DEGs). The distribution of differentially expressed genes (DEGs) in different combinations of roots of *Euphorbia angustifolia* at different time (0 d, 1 d, 3 d, 7 d) of treatment group (AL1_400, AL3_400, AL7_400) and control group (AL0, AL1_CK, AL3_CK, AL7_CK). The Abscissa represents the multiple change of gene expression in different samples; the ordinate indicates the statistically significant difference in gene expression; the red dot in the picture indicates the up-regulated gene with significant difference in expression, and the green dot represents the down-regulated gene with significant difference in expression. (FDR < 0.05, $|\log_2(\text{FoldChange})| > 0$).

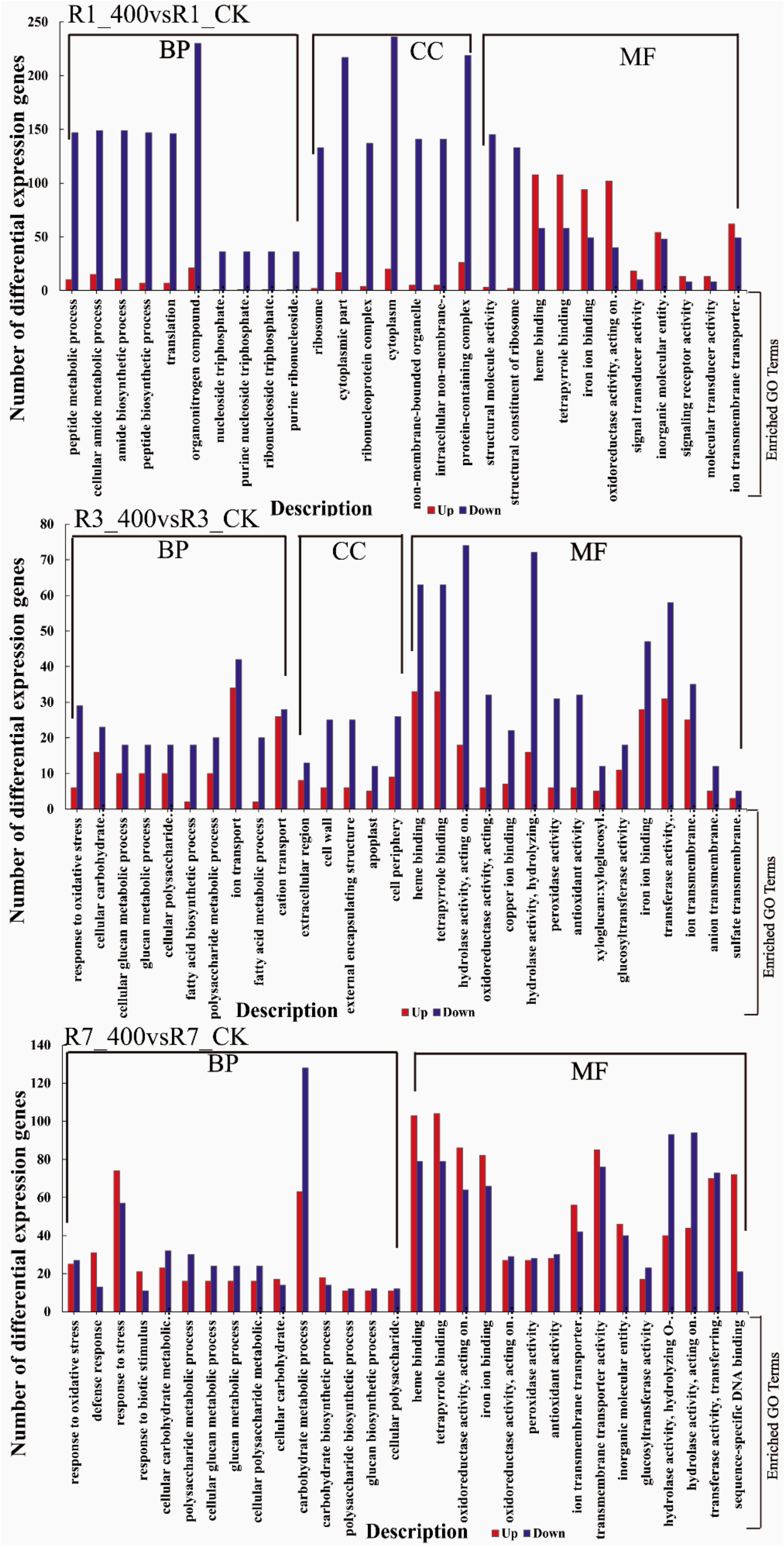

Figure Supplementary 3. GO enrichment of three comparative combinations of aluminum stress in different periods. The DEGs in the first 10 pathways that were significantly enriched in different periods of aluminum stress were screened, and all DEGs in less than 10 pathways were displayed. The abscissa represents the different GO term enriched genes, the ordinate represents the number of

DEGs. The red bar represents the up-regulated genes, the blue bar represents the down-regulated genes. BP represents DEGs enriched in biological processes, MF represents DEGs enriched in molecular functions, and CC represents DEGs enriched in cellular component.

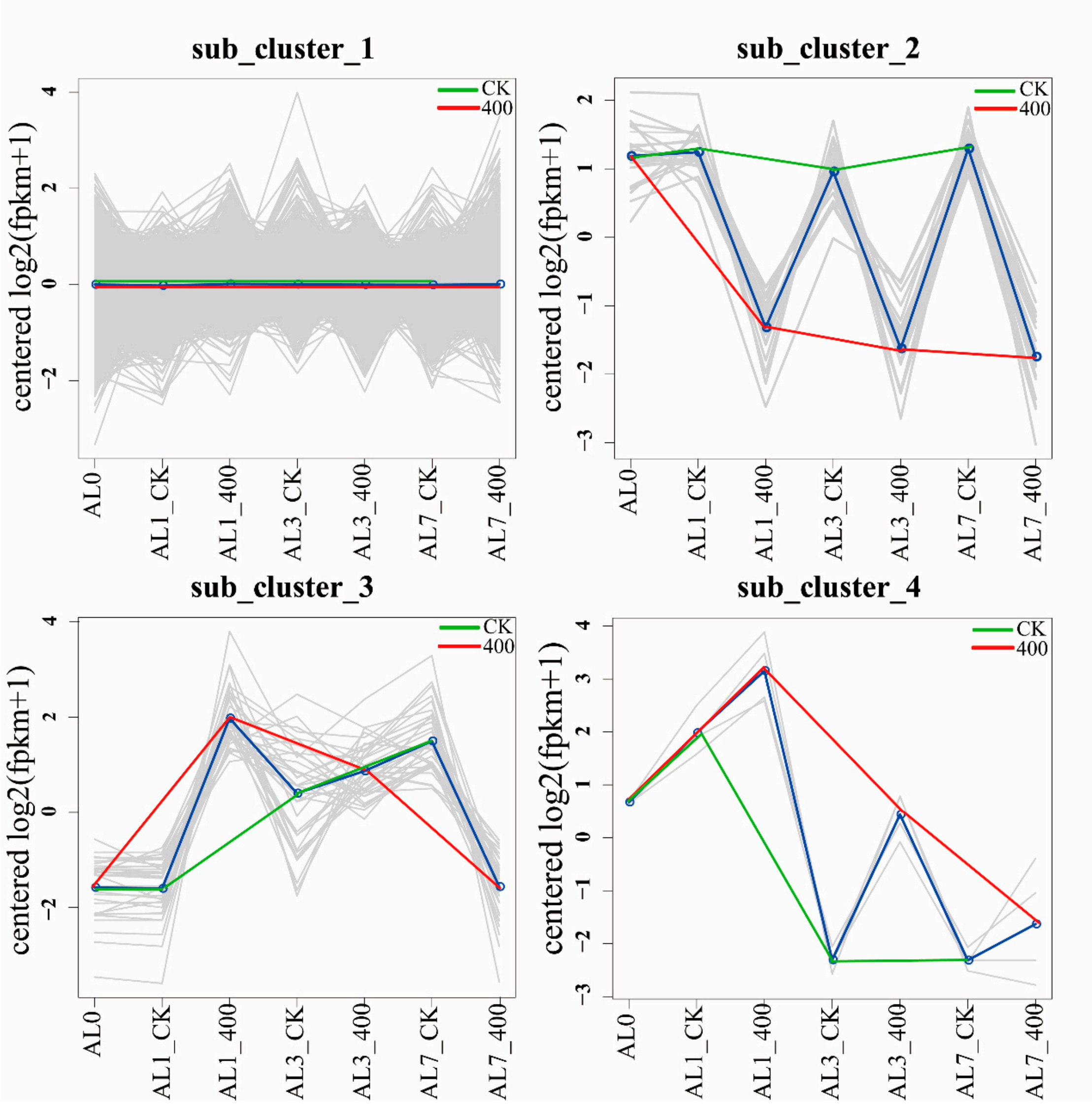

Figure Supplementary 4. Trend clustering of DEGs. The green lines indicate the trend of gene clustering in the control group; the red lines indicate the trend of gene clustering in the treatment group; the blue line shows the average relative expression of all genes in this cluster under different experimental conditions; The gray lines in each subgraph represent a line chart of the relative expression of genes in a cluster under different experimental conditions. .

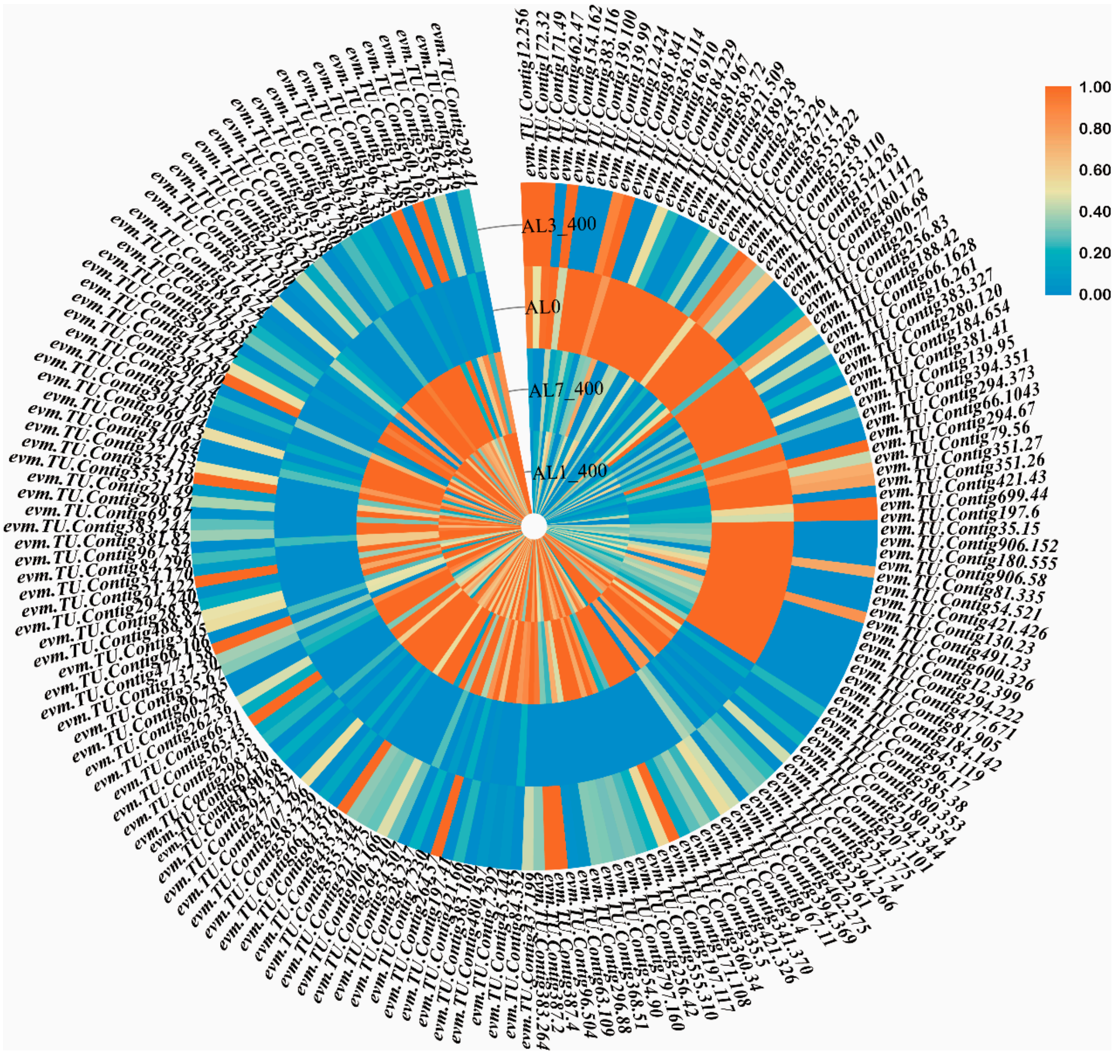

Figure Supplementary 5. Heat map of DEGs expression in response to aluminum stress in various periods. The FDR (FDR < 0.05) and the fold change (|log_2(FoldChange)| > 1) were used as the differential gene screening threshold to compare the changes of differential gene expression between different the treatment groups and control group on the 1st day.

Table Supplementary 1. Quality evaluation of sequencing data

**Sample name**	**Raw reads**	**Clean reads**	**clean bases**	**Error rate(%)**	**Q20(%)**	**Q30(%)**	**GC content(%)**
AL0_1	61194292	60187436	9.03G	0.02	98.36	94.76	43.64
AL0_2	68003714	66818940	10.02G	0.02	98.17	94.29	43.69
AL0_3	63752868	62619194	9.39G	0.02	98.41	94.88	43.58
AL1_CK_1	68026854	66863458	10.03G	0.02	98.3	94.58	43.52
AL1_CK_2	60377232	59463000	8.92G	0.02	98.26	94.51	43.58
AL1_CK_3	55716572	54928798	8.24G	0.03	98.03	93.92	43.53
AL1_400_1	70108628	68863288	10.33G	0.03	96.8	91.39	43.33
AL1_400_2	56905498	56051598	8.41G	0.02	98.28	94.59	43.45
AL1_400_3	66808072	65804690	9.87G	0.03	96.73	91.26	43.32
AL3_CK_1	60255320	59122780	8.87G	0.02	98.27	94.52	43.66
AL3_CK_2	51855712	50304222	7.55G	0.02	98.25	94.47	43.66
AL3_CK_3	55026252	53363844	8.0G	0.03	96.96	91.71	43.44
AL3_400_1	56693196	55530186	8.33G	0.02	98.31	94.62	43.55
AL3_400_2	61127434	59615668	8.94G	0.03	97.37	92.49	43.41
AL3_400_3	60287314	59056562	8.86G	0.02	98.4	94.87	43.55
AL7_CK_1	64215260	62625458	9.39G	0.02	98.27	94.51	43.49
AL7_CK_2	54868194	52896640	7.93G	0.03	96.98	91.72	43.4
AL7_CK_3	64744542	63356668	9.5G	0.03	98.06	93.98	43.65
AL7_400_1	66694232	65272288	9.79G	0.02	98.45	94.95	44.23
AL7_400_2	46326344	43991788	6.6G	0.02	98.45	95.03	43.73
AL7_400_3	66692556	65368558	9.81G	0.02	98.29	94.52	43.78

The number of reads in the raw data; clean_reads：the number of reads filtered from the original data; clean_bases：the number of bases filtered from the original data; error_rate：error rate of overall data sequencing; Q20：the percentage of bases with a Phred value greater than 20 to the total base; Q30：the percentage of

bases with a Phred value greater than 30 in the total base; GC content： the percentage of G and C in four bases in clean reads.

Table Supplementary 2. Statistics on the alignment of lean reads and reference genomes

**Sample name**	**total_reads**	**total_map**	**unique_map**	**multi_map**	**read1_map**	**read2_map**
AL0_1	60187436	56418328(93.74%)	54541126(90.62%)	1877202(3.12%)	27323351(45.4%)	27217775(45.22%)
AL0_2	66818940	62458819(93.47%)	60330247(90.29%)	2128572(3.19%)	30256890(45.28%)	30073357(45.01%)
AL0_3	62619194	58666811(93.69%)	56687286(90.53%)	1979525(3.16%)	28388327(45.33%)	28298959(45.19%)
AL1_CK_1	66863458	63613029(95.14%)	61621016(92.16%)	1992013(2.98%)	30873659(46.17%)	30747357(45.99%)
AL1_CK_2	59463000	55711716(93.69%)	53945557(90.72%)	1766159(2.97%)	27030193(45.46%)	26915364(45.26%)
AL1_CK_3	54928798	51970207(94.61%)	50381570(91.72%)	1588637(2.89%)	25283906(46.03%)	25097664(45.69%)
AL1_400_1	68863288	64624456(93.84%)	62560046(90.85%)	2064410(3.0%)	31438883(45.65%)	31121163(45.19%)
AL1_400_2	56051598	53231186(94.97%)	51505558(91.89%)	1725628(3.08%)	25810115(46.05%)	25695443(45.84%)
AL1_400_3	65804690	61894880(94.06%)	59857820(90.96%)	2037060(3.1%)	30074252(45.7%)	29783568(45.26%)
AL3_CK_1	59122780	56202972(95.06%)	54270913(91.79%)	1932059(3.27%)	27201549(46.01%)	27069364(45.78%)
AL3_CK_2	50304222	47146214(93.72%)	45595077(90.64%)	1551137(3.08%)	22851487(45.43%)	22743590(45.21%)
AL3_CK_3	53363844	49968720(93.64%)	48323788(90.56%)	1644932(3.08%)	24267845(45.48%)	24055943(45.08%)
AL3_400_1	55530186	52779055(95.05%)	51110254(92.04%)	1668801(3.01%)	25601973(46.1%)	25508281(45.94%)
AL3_400_2	59615668	56260050(94.37%)	54480814(91.39%)	1779236(2.98%)	27315745(45.82%)	27165069(45.57%)
AL3_400_3	59056562	55828623(94.53%)	54050775(91.52%)	1777848(3.01%)	27061792(45.82%)	26988983(45.7%)
AL7_CK_1	62625458	58136633(92.83%)	56212441(89.76%)	1924192(3.07%)	28166155(44.98%)	28046286(44.78%)
AL7_CK_2	52896640	49258323(93.12%)	47633351(90.05%)	1624972(3.07%)	23889000(45.16%)	23744351(44.89%)
AL7_CK_3	63356668	59346469(93.67%)	57330146(90.49%)	2016323(3.18%)	28760173(45.39%)	28569973(45.09%)

Total_reads： clean reads number of sequencing data after quality control; total_map： compare the number and percentage of reads on the genome; multi_map：the number and percentage of reads mapped to multiple locations of the reference genome; unique_map: the number and percentage of reads compared to the unique location of the reference genome; read1_map：the number and percentage of read1 compared to the reference genome; read2_map：the number and percentage of read2 compared to the reference genome.

Table Supplementary 3. Primer pairs used to verify gene fluorescence quantification

Genes	Forward Primer (5' - 3')	Reverse Primer (5'- 3')
evm.TU.Contig491.4	CTTGGCTCCGTTGTTGCTTG	AAGTTGCCCCTCTTTGGGTC
evm.TU.Contig368.51	AGGCAGCATTGAACCCTGAT	AGCCCTCATCCTGCCAATTC
evm.TU.Contig81.157	TGCGAAAAGCGACAAATCCC	CCACTGCTGGAGAACCAAGT
evm.TU.Contig583.176	TTTGTGGCCTTGAGTTCCCC	ACGTTCGTTACGGTGTCGTC
evm.TU.Contig207.236	CATCGGGTGGTCGTGTTTTC	CATGCTTCTTAGGCTTCCGC
evm.TU.Contig63.553	TCCCGTTGCAGGAATGATGG	CACTGGGGACAGGGCAATAA
evm.TU.Contig154.214	ACCGCCTTGAAATCCCTTCC	AAGCGGTACAATTCGTGGCT
evm.TU.Contig154.133	ACTTCAGCTTCCAGCATCCC	CAATGCCGTATGCACAGTCG
evm.TU.Contig66.895	CCTGATTCTGTCCGAGCCAT	TGTTGGCACCCTTTTCGTCT
evm.TU.Contig519.36	ACAGAAAAGGATGGTGGCAGA	TGCCAAAGGACAGTGATCCG
evm.TU.Contig21.81	AAATGGACCGACCCGCATAG	AACGCTCTTGTAATTGCGCC
evm.TU.Contig488.39	CCGACATCCTTGGGGACAAT	AGGGAGTCAAGACGATAGGCA
evm.TU.Contig171.52	GGTGCCTTTGCTTTGCATGA	TGGTGATGATGGTGCTTTCCA
evm.TU.Contig96.340	TTGAAGCAACTACCCCGACC	TTGCGTCTCAGCCTTCAGTT
evm.TU.Contig948.4	AGTGGACGAGGTATCGAGGT	GCCCGATCATAAGCCAATGC
evm.TU.Contig151.18	CAGAGTGGTCGGATTGCCTC	GCACGTAGCACACGTTACAG
evm.TU.Contig84.515	CCCCCGATCTTCCTTTTGCT	TGGTCTCATTGGGCATGAAGT

Table Supplementary 4. High expression of transporters-encoding and protein kinases encoding genes

Gene_id		log$_2$FoldChange			Gene Name
		**AL1_400vsAL1_CK**	**AL3_400vsAL3_CK**	**AL7_400vsAL7_CK**	
	**up**	25	29	28	
	**down**	21	18	19	
**Transporter**	*evm.TU.Contig14.261*	-0.121622481	-1.44918	-1.24121	ABCB13
	evm.TU.Contig383.77	1.758411228	0.682502	2.997459	ABCB15
	evm.TU.Contig66.296	-0.280714207	0.976585	2.446902	ABCB19
	evm.TU.Contig98.26	1.34272343	0.644218	3.402411	ABCB9
	evm.TU.Contig1.102	0.587820918	1.885543	2.940956	ABCC2
	evm.TU.Contig341.248	-0.332416975	-1.36422	-0.82942	ABCC5
	evm.TU.Contig967.193	0.841170134	0.870238	1.34585	ABCC8
	evm.TU.Contig184.120	-0.134403036	0.333368	-1.39083	ABCC9
	evm.TU.Contig583.11	-0.31016166	-1.13194	-0.45071	ABCF1
	evm.TU.Contig16.373	-1.028430204	-3.20704	-2.57766	ABCG10
	evm.TU.Contig694.215	-1.685158875	4.033051	3.007732	ABCG11
	evm.TU.Contig486.7	1.15134808	0.861225	1.343725	ABCG14
	evm.TU.Contig66.774	2.480825243	-0.36736	0.037733	ABCG17
	evm.TU.Contig184.885	-0.71639939	-0.95938	-1.56852	ABCG21
	evm.TU.Contig468.8	-0.077036887	-1.17795	-0.40441	ABCG23
	evm.TU.Contig81.396	0.025017427	-0.51158	-1.36486	ABCG24
	evm.TU.Contig63.68	1.015241478	0.853779	0.594683	ABCG30
	evm.TU.Contig180.280	-0.933270576	0.59209	1.051397	ABCG32
	evm.TU.Contig63.72	-0.130994072	-0.39352	-1.15944	ABCG40
	evm.TU.Contig860.17	-1.990284054	-3.56959	-3.42524	ABCG40
	evm.TU.Contig81.1119	-1.345854341	0.307669	1.156213	ABCG6

	evm.TU.Contig860.19	-0.449622089	1.358583	1.705097	ABCI20
	evm.TU.Contig477.252	1.327312091	-0.40422	-0.59885	ALMT10
	evm.TU.Contig154.52	2.823423011	1.096949	2.266489	ALMT10
	evm.TU.Contig969.22	-0.937404989	1.595426	1.68336	ALMT12
	evm.TU.Contig63.261	4.355505406	0.880934	5.371157	ALMT2
	evm.TU.Contig12.396	1.103505998	1.5693	3.211727	SLC47A1
	evm.TU.Contig84.309	0.463048282	-4.66038	-5.51479	POT2
	evm.TU.Contig421.490	1.531208376	-0.54127	-0.42175	POT5
	evm.TU.Contig69.71	-0.601655184	1.548719	1.581654	POT5
	evm.TU.Contig694.68	-0.307176684	-1.77064	-1.39112	At1g71900
	evm.TU.Contig437.189	-0.779874862	-1.24489	-0.87962	At5g11960
	evm.TU.Contig166.25	1.581465915	5.447464	-0.03108	ZIP10
	evm.TU.Contig16.637	0.411911777	-1.22009	-1.18417	ABCB8
	evm.TU.Contig765.15	-0.634892748	-1.09606	-1.33446	At1g54730
	evm.TU.Contig5.75	2.163089269	0.195573	0.055891	ZIP1
	evm.TU.Contig139.260	0.551929662	0.9105	1.330903	ZIP2
	evm.TU.Contig5.266	0.206621403	1.332927	0.261797	ZIP4
	evm.TU.Contig672.2	-0.317357347	1.958847	1.678213	ZIP5
	evm.TU.Contig481.169	0.811530726	1.179202	2.72664	ZIP5
**protein kinase**	*evm.TU.Contig141.53*	4.12081016	-0.52841	-0.66694	SOBIR1
	evm.TU.Contig141.52	3.203429024	0.312604	0.094061	SOBIR1
	evm.TU.Contig81.601	0.272979453	0.885326	1.633123	MAKR4
	evm.TU.Contig60.245	0.163565932	0.666918	1.227542	PKS1
	evm.TU.Contig130.60	/	3.901552	5.317494	PKS3
	evm.TU.Contig201.48	0.38196684	1.543087	1.988568	At3g51990
	evm.TU.Contig766.72	-0.632039789	1.934396	1.290876	CCR1

The FDR (FDR < 0.05) and the fold change ($|\log_2(\text{FoldChange})| > 1$) were used as indicators to screen the differentially expressed genes between aluminum stress induced group and control group at different time. A negative value of $\log_2$FoldChange indicates that the gene expression is down-regulated, while a positive value indicates that the gene expression is up-regulated.

Table Supplementary 5. New DEGs and unnamed DEGs

Gene ID		log₂FoldChange			Description
		R1_400vsR1_CK	R3_400vsR3_CK	R7_400vsR7_CK	
	up	25	24	23	
	down	52	53	54	
evm.TU.Contig12.254		-1.68032	-2.13683	-2.21447	unnamed protein product
evm.TU.Contig139.44		-1.34723	-2.34128	-1.78706	unnamed protein product
evm.TU.Contig14.145		-1.14208	-2.13592	-1.50448	unnamed protein product
evm.TU.Contig14.325		-1.76456	-1.53771	-3.0853	unnamed protein product
evm.TU.Contig14.326		-0.97391	-1.42962	-2.0822	unnamed protein product
evm.TU.Contig143.12		-3.96174	-3.55446	-3.30114	unnamed protein product
evm.TU.Contig151.66		1.95868	1.968553	3.56543	unnamed protein product
evm.TU.Contig154.193		0.885835	2.214445	1.86615	unnamed protein product
evm.TU.Contig154.659		-2.10398	-3.58933	-1.97985	unnamed protein product
evm.TU.Contig184.424		1.205629	2.006807	2.147204	unnamed protein product
evm.TU.Contig184.57		-1.21883	-1.7173	-2.25449	unnamed protein product
evm.TU.Contig184.586		-2.91187	-5.80906	-3.80385	unnamed protein product
evm.TU.Contig21.498		2.137926	1.664477	2.31157	unnamed protein product
evm.TU.Contig256.6		2.115634	1.781262	2.497298	unnamed protein product
evm.TU.Contig271.70		1.664998	1.583371	2.536936	unnamed protein product
evm.TU.Contig271.71		1.371642	1.626755	2.279892	unnamed protein product
evm.TU.Contig280.67		2.241073	1.593307	1.684287	unnamed protein product
evm.TU.Contig296.151		-4.90499	-5.46567	-6.7321	unnamed protein product
evm.TU.Contig368.46		-2.74083	-1.79621	-3.24205	unnamed protein product
evm.TU.Contig368.47		-2.76407	-2.12096	-3.27932	unnamed protein product
evm.TU.Contig447.157		0.96409	-3.35752	-3.48368	unnamed protein product
evm.TU.Contig45.123		-1.34708	-2.3494	-1.53932	unnamed protein product
evm.TU.Contig471.132		-1.23449	-2.04507	-1.51728	unnamed protein product

evm.TU.Contig477.396	-2.20047	-3.70606	-2.90498	unnamed protein product
evm.TU.Contig477.721	-1.2679	-2.03637	-2.47889	unnamed protein product
evm.TU.Contig480.386	-3.53075	-1.48989	-5.31566	unnamed protein product
evm.TU.Contig55.215	1.811465	1.525186	2.064504	unnamed protein product
evm.TU.Contig555.77	-2.06753	-1.84046	-2.54481	unnamed protein product
evm.TU.Contig586.8	2.335668	1.465431	3.197961	unnamed protein product
evm.TU.Contig60.474	-0.88135	-2.06653	-1.52982	unnamed protein product
evm.TU.Contig600.222	3.816377	2.304929	2.871286	unnamed protein product
evm.TU.Contig600.57	2.846669	2.078649	2.785662	unnamed protein product
evm.TU.Contig63.371	-1.09605	-1.39256	-2.21673	unnamed protein product
evm.TU.Contig66.311	-1.65001	-1.55028	-2.78768	unnamed protein product
evm.TU.Contig66.781	3.421413	2.077415	1.927894	unnamed protein product
evm.TU.Contig797.219	2.940478	3.055996	3.768322	unnamed protein product
evm.TU.Contig797.67	-1.91577	-2.37416	-2.35697	unnamed protein product
novel.1026	-1.39844	-2.81986	-1.71505	new gene
novel.1040	3.026199	1.250311	1.817744	new gene
novel.109	-1.17115	-3.1877	-1.83652	new gene
novel.116	1.433453	1.243581	2.851857	new gene
novel.122	-3.12123	-3.16949	-3.65924	new gene
novel.125	-2.00935	-1.80948	-3.95399	new gene
novel.1308	-6.09709	-8.55061	-5.96819	new gene
novel.1325	-3.31625	-4.41164	-2.36392	new gene
novel.1326	-2.16862	-2.51953	-3.22303	new gene
novel.1344	-1.08814	-3.7315	-2.47517	new gene
novel.138	-2.31072	-1.97709	-3.20647	new gene
novel.1618	-4.35506	-2.62978	-2.84897	new gene
novel.1777	-1.72736	-0.88269	-2.28467	new gene
novel.1778	-5.38369	-3.85791	-5.0134	new gene
novel.1784	-6.17447	-4.77418	-5.08971	new gene

novel.1834	5.237935	3.577414	5.365558	new gene
novel.1839	4.42903	2.824671	5.120345	new gene
novel.1846	-1.53519	-2.47915	-1.61022	new gene
novel.1871	5.608535	3.67216	-3.68213	new gene
novel.1912	-1.49282	-2.05183	-2.27075	new gene
novel.21	-1.87753	-2.14708	-3.28464	new gene
novel.278	1.612914	1.426325	2.825292	new gene
novel.438	7.140842	3.507089	4.615261	new gene
novel.54	-0.67544	-2.558	-1.16692	new gene
novel.547	-4.45889	-4.23123	-2.71747	new gene
novel.548	-3.11392	-2.85048	-3.11139	new gene
novel.56	-4.06775	-3.4372	-6.07657	new gene
novel.58	-2.39682	-2.12457	-2.99169	new gene
novel.59	-1.33916	-2.92269	-1.92548	new gene
novel.60	-2.89228	-4.39289	-4.30914	new gene
novel.619	-1.11134	-2.20149	-3.84177	new gene
novel.623	-2.06662	-2.57085	-1.71939	new gene
novel.639	2.326975	1.25429	0.79408	new gene
novel.688	-2.3588	-3.48776	-2.41744	new gene
novel.811	2.623409	1.559927	1.512646	new gene
novel.861	-1.82287	-2.25526	-2.4827	new gene
novel.901	2.084617	1.851716	2.822601	new gene
novel.938	-2.11981	-2.07641	-1.17352	new gene
novel.939	-1.4746	-2.11379	-1.13413	new gene
novel.989	-4.29487	-6.88285	-10.5275	new gene

	R1_400vsR0	R3_400vsR1_400	R7_400vsR3_400
up	25	24	23
down	52	53	54

evm.TU.Contig16.712	2.380067495	-1.49085964	1.20212316	unnamed protein product

evm.TU.Contig318.21	-2.189921877	1.4118865	-1.799735417	unnamed protein product
evm.TU.Contig480.393	2.941809	-0.53452	-1.47162	unnamed protein product
evm.TU.Contig60.166	2.027039	0.57279	-0.82097	unnamed protein product
evm.TU.Contig600.222	2.635218	1.532061	1.24221	unnamed protein product
evm.TU.Contig66.901	2.661035	0.498963	-1.03932	unnamed protein product
evm.TU.Contig81.474	3.843881	-0.64851	-1.33899	unnamed protein product
evm.TU.Contig84.318	1.891238	0.93173	-2.69735	unnamed protein product
evm.TU.Contig906.35	4.123436	-0.88732	-2.37734	unnamed protein product
novel.120	-3.28835	2.874898	-3.67847	new gene
novel.1219	-2.00927	1.699088	-2.4735	new gene
novel.1431	5.464379	3.402045	1.979997	new gene
novel.482	-3.46163	1.507224	-2.31423	new gene
novel.619	-1.52041	-0.87076	-2.06521	new gene
novel.950	2.785206	2.367055	1.916898	new gene
novel.976	-1.70888	2.00455	-0.96501	new gene

The FDR (FDR < 0.05) and the fold change ($|\log_2(\text{FoldChange})| > 1$) were used as indicators to screen the differentially expressed genes after aluminum stress in contrast to control conditions. A negative value of $\log_2$FoldChange indicates that the gene expression is down-regulated, while a positive value indicates that the gene expression is up-regulated.
